# An overview of cutaneous T cell lymphomas

**DOI:** 10.12688/f1000research.8829.1

**Published:** 2016-07-28

**Authors:** Nooshin Bagherani, Bruce R. Smoller

**Affiliations:** 1 Taha Physicians' Building, Khoramshahr, Khuzestan Province, Iran; 2 Department of Dermatology, School of Medicine and Dentistry, University of Rochester, Rochester, New York, USA

**Keywords:** cutaneous T cell lymphoma, mycosis fungoides, Sézary syndrome

## Abstract

Cutaneous T cell lymphomas (CTCLs) are a heterogeneous group of extranodal non-Hodgkin’s lymphomas that are characterized by a cutaneous infiltration of malignant monoclonal T lymphocytes. They typically afflict adults with a median age of 55 to 60 years, and the annual incidence is about 0.5 per 100,000. Mycosis fungoides, Sézary syndrome, and primary cutaneous peripheral T cell lymphomas not otherwise specified are the most important subtypes of CTCL. CTCL is a complicated concept in terms of etiopathogenesis, diagnosis, therapy, and prognosis. Herein, we summarize advances which have been achieved in these fields.

## Introduction

In 1806, Alibert initially described mycosis fungoides (MF) as the infiltration of skin by lymphocytes. In 1974, Edelson used the term “cutaneous T cell lymphomas” (CTCLs) for MF and its leukemic variant, Sézary syndrome (SS), which are the major types of CTCL ^1^. Nowadays, the CTCLs, which are characterized by infiltration of malignant monoclonal T lymphocytes in the skin, are considered a heterogeneous group of extranodal non-Hodgkin’s lymphomas ^1– 3^. Approximately 25% to 40% of non-Hodgkin’s lymphoma cases involve extranodal sites. The skin is the most common site after the gastrointestinal system ^4^. The annual incidence of CTCL is about 0.5 per 100,000, and men are more involved than women (1.6:1 to 2.0:1) ^1^. They typically afflict adults with a median age of 55 to 60 years ^1, 5^.

In 1975, for the first time, the North American Mycosis Fungoides Cooperative Study Group classified the CTCLs on the basis of a tumor-node-metastasis (TNM) system. Thereafter, the classification was modified and updated by the CTCL workshop to the one used today, known as the Bunn and Lambert system ^1^. The International Society for Cutaneous Lymphomas/European Organization for Research and Treatment of Cancer classified MF and SS according to clinical, pathological, biological, and immunological features ^6^.

**Figure 1.**
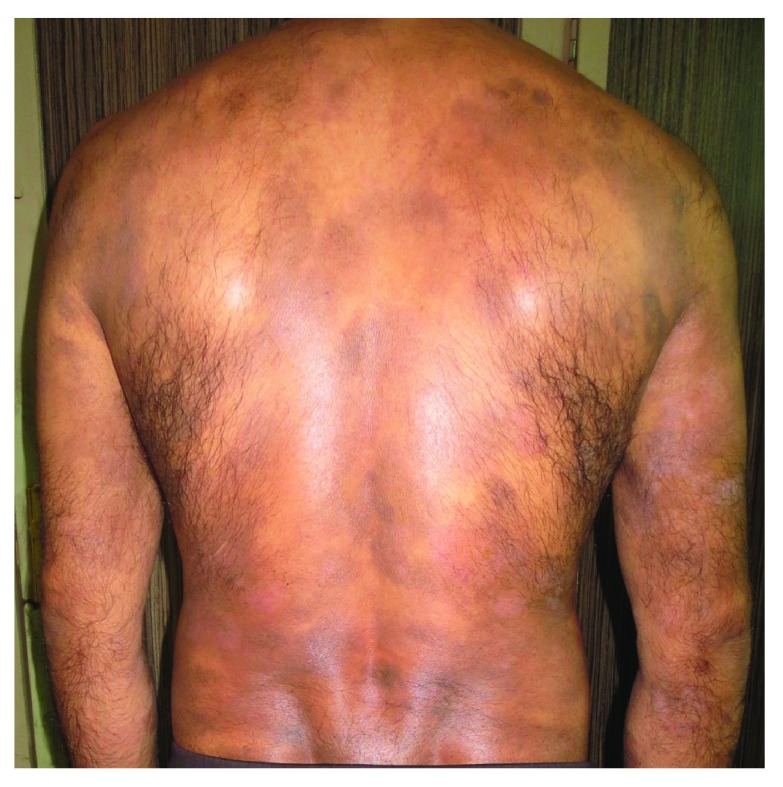
Mycosis fungoides in a 40-year-old man manifested as generalized atrophic patches.

MF, SS, and primary cutaneous peripheral T cell lymphomas not otherwise specified (PCTCL - NOS) are among the most important subtypes of the CTCLs ^7, 8^. MF is the commonest type of CTCLs, representing 44% to 62% of cases ^9^. MF restricted to the skin has an indolent progression passing from macule and patch stage to infiltrated plaque and tumor stage ( Figure 1) ^5, 10, 11^ in sun-protected body sites ^12^. SS is defined as an aggressive leukemic-phase type of MF ^11^, clinically characterized by erythroderma and generalized lymphadenopathy ^13^. PCTCL-NOS can present with a solitary red-violaceous tumor-like nodule or scattered multifocal or diffuse nodules on any part of the body, which mostly become ulcerated and infected. Rapid cutaneous dissemination and systemic involvement are key features of this class of CTCL ^8^.

At the early stages, CTCLs are often misdiagnosed as benign skin conditions ^1, 9, 13– 17^. Their most important differential diagnoses have been listed in Table 1.

**Table 1.** The most important differential diagnoses of the cutaneous T cell lymphomas.

All kinds of dermatitis and eczema ^1, 9, 13– 15^ Adverse drug reactions ^13^ Parapsoriasis ^9^ Psoriasis ^1, 14, 15^ Lichen planus ^16^ Morphea ^16^ Panniculitis ^17^ Folliculitis ^14^ Pityriasis lichenoides chronica ^14^ Pityriasis lichenoides et varioliformis acuta ^14^ Pigmented purpuric dermatoses ^14^ Vitiligo ^14^ Lymphomatoid papulosis ^9^

**Figure 2.**
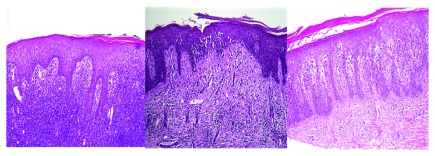
In pathological view, the cutaneous T cell lymphomas are characterized by haloed lymphocytes, exocytosis, epidermotropism, Pautrier’s microabscess, large hyperconvoluted, hyperchromatic lymphocytes in the epidermis, and lymphocytes aligned within the basal layer. **Figure 2A** (left) A lymphocytic infiltrate is present in the dermis and extending into the overlying epidermis with minimal overlying spongiosis. **Figure 2B** (center) Lymphocytes with surrounding haloes are present in the epidermis as single cells and small clusters (Pautrier’s microabscesses). There is minimal accompanying spongiosis. **Figure 2C** (right) Psoriasiform epidermal hyperplasia with epidermotropism of haloed lymphocytes is seen in this case of patch-stage mycosis fungoides.

In most patients with CTCL, the histologic features are subtle, so that the differentiation of these disorders from benign inflammatory diseases is difficult ^18, 19^. Haloed lymphocytes, exocytosis, epidermotropism, Pautrier’s microabscess, large hyperconvoluted, hyperchromatic lymphocytes in the epidermis, and lymphocytes aligned within the basal layer are findings seen in histologic sections of MF ( Figure 2) ^16, 20^.

CTCL is a complicated concept in terms of etiopathogenesis, diagnosis, therapy, and prognosis. Herein, we have summarized advances which have been achieved in these fields.

## Etiopathogenesis

**Figure 3.**
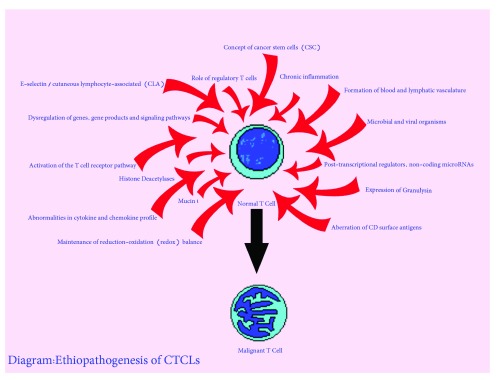
Etiopathogenesis of cutaneous T cell lymphoma. CTCL, cutaneous T cell lymphoma.

Although different views of CTCL etiopathogenesis have been elucidated in depth over the last few decades, the exact mechanism of initiation and progression of this disorder is not yet known ^4, 10, 21^ ( Figure 3). Although dysregulation of some genes and signaling pathways has been reported in the CTCLs ( Table 2), their exact role in the pathogenesis of these disorders is unknown ^2, 3, 13, 22– 34^. Most of these aberrations are seen in chromosome 10 ^35^. Recent studies demonstrate ectopic expression of cancer testis genes in the CTCLs. It appears that this gene works through inhibiting apoptosis, inducing resistance to various forms of therapeutic modalities, and contributing to oncogenesis by targeting tumor suppressor genes such as *p53* and *p21* ^2^. Deficient expression or function of negative regulators, including SOCS3 and protein tyrosine phosphatases such as SHP1, have been implicated in dysregulation of the Jak-3/STAT pathway and interleukin (IL)-independent proliferation of malignant T cells. The Jak-3/STAT pathway has a role in fighting against CTCLs by promoting production of IL-5, IL-10, IL-17A, and IL-17F; regulating angiogenic factors; and interfering with resistance to histone deacetylase inhibitor (HDACI) therapy ^25^. An additional report demonstrates pathogenic involvement of the NOTCH1 signaling pathway in the pathogenesis of SS ^13^. NOTCH includes a family of transmembrane receptors, which play roles in cell differentiation, proliferation, and stemness ^31^.

**Table 2.** An alphabetical list of dysregulation of genes and signaling pathways seen in the cutaneous T cell lymphomas.

AT-rich interactive domain-containing protein 1A (ARID1A) ^23^ B-Raf proto-oncogene, serine/threonine kinase (BRAF) ^24^ Bromodomain-9 (BRD-9) ^24^ Cancer testis (CT) ^2^ Caspase recruitment domain (CARD11) ^23, 24^ C-C chemokine receptor-4 (CCR-4) ^23^ Chromodomain-helicase-DNA-binding protein (CHD)-3 ^24^ CREB-binding protein (CREBBP) ^24^ Cyclin-dependent kinase-2 (CDKN-2) ^23– 26^ Cyclooxygenase-2 (COX-2) ^25^ Dynamin-3 (DNM-3) ^22^ Embryonic stem cell regulators ^3, 25^ Eph receptor A4 (EPHA4) ^13^ Forkhead box P3 (FOXP3) ^27^ GATA-binding protein-3 (GATA-3) ^22, 25^ Histone deacetylase-6 (HDAC-6) ^25^ Histone-lysine N-methyltransferase (KMT)-2D or myeloid/lymphoid or mixed-lineage leukemia protein-2 (MLL-2) ^24^ Interleukin-2 receptor common gamma chain (IL-2Rgc) ^3, 25^ Janus kinase-3 (Jak-3)/signal transducers and activators of transcription (STAT) ^25, 28^ KIRD3DL2 ^22^ Lysine (K)-specific methyltransferase (KMT)-2C or myeloid/lymphoid or mixed-lineage leukemia protein-3 (MLL-3) ^24^ MYC-associated factor X (MAX) ^25^ Metallothioneins I and II (MTI/II) ^29^ Methylthioadenosine-phosphorylase (MTAP) ^30^ Mitogen-activated protein kinase 1 (MAPK1) ^24^ MYC-binding protein (MYCBP) ^25^ NOTCH1 ^13, 31^ Nuclear factor kappa-light-chain-enhancer of activated B cells (NFκB) ^3, 24, 25^ Nuclear factor of activated T cells (NFAT) ^24^ Phospholipase C gamma 1 (PLCG1) ^23, 32^ P21 ^33^ P53 ^28, 32^ Phosphatase and tensin homolog (PTEN) ^24, 25, 34^ Plastin-3 (PLS-3) ^22^ Protein kinase, CGMP-dependent (PRKG1) ^24^ Receptor tyrosine kinase (RTK) ^13^ Retinoblastoma-1 (RB-1) ^24^ Ribosomal protein S6 kinase-1 (RPS6KA-1) ^23^ Runt-related transcription factor-3 (RUNX-3) ^22^ Src homology region 2 domain-containing phosphatase-1 (SHP1) ^25^ Special AT-rich sequence-binding protein 1 (SATB1) ^29^ SRC proto-oncogene, non-receptor tyrosine kinase (Src kinases) ^25^ Suppressors of cytokine signaling-3 (SOCS3) ^25^ SWI/SNF-related, matrix-associated, actin-dependent regulator of chromatin-A4 (SMARC-A4) ^24^ Tet methylcytosine dioxygenase 2 (TET2) ^24^ Thymocyte selection-associated high-mobility group box (TOX) ^22, 25^ Tumor protein p53 (TP53) ^23, 24^ Tumor necrosis factor receptor superfamily-1B (TNFRSF-1B) ^30^ Twist ^22^ Zinc finger E-box-binding homeobox 1 (ZEB1) ^23^

The loss of heterozygosity of phosphatase and tensin homolog (PTEN) in MF has been reported, but the significance of the finding remains unclear. In one study, a statistically significant decrease in the percentage of cells retaining PTEN and altered staining intensity were shown in MF from patch to plaque stage, but this attenuation was not significant in tumor stage in comparison with plaque stage ^32^. The mRNA expression of a subfamily of receptor tyrosine kinase (RTK) is high in SS but not in other types of CTCLs ^13^.

TOX is a transcription factor with a role in the development of CD4 ^+^ T cells, including downstream effects on the expression of *RUNX 3*, a well-known tumor suppressor gene. Studies show that the overexpression of TOX and its protein product is associated with thicker lesions of MF, disease progression, and poor prognosis. Furthermore, dysregulation of this gene is reported in lesions and peripheral blood mononuclear cells of cases with SS ^22^.

Patients with CTCL show a distinct microRNA (miRNA) expression profile. Studies have shown that miR-21 and miR-155 are associated with poor prognosis and aggressive behavior by interfering with resistance to apoptosis and promoting malignant proliferation, respectively. On the other hand, the expression of miR-22, a tumor suppressor, is downregulated in SS. It appears that Jak-3/STAT is responsible for the loss of miR-22 expression ^25^. miR-16 is another non-coding microRNA that induces cellular senescence and is downregulated in CTCLs ^33^. Studies have shown that miRNAs inhibit the expression of many oncogenes such as *MAX*, *MYCBP*, nuclear receptor coactivator-1 ( *NCOA-1*), and cyclin-dependent kinase-6 ( *CDK-6*) ^25^.

A role for IL-2Rgc-signaling cytokines, including IL-2, IL-4, IL-7, IL-15, and IL-21, has been suggested in the pathogenesis of CTCLs ^25^. IL-12 plays a role as a potent anti-tumor agent. Its expression is decreased during tumoral-stage MF ^36^. On the other hand, in lesional skin of CTCLs, increased expression of IL-9 that is regulated by STAT3/5 and silencing of STAT5 has been reported ^37^. Additionally, upregulation of CC chemokine receptor 6 (CCR6) ^38^ and CCR7 ^3^ has been reported in CTCLs and alleged to be responsible for spreading malignant T cells to sentinel lymph nodes, the bloodstream, and internal organs. Chemokine (C-X-C motif) ligand 12 (CXCL12) belongs to the superfamily of chemokines that is expressed on endothelial and stromal cells in different organs. Most hematopoietic cells such as CD34 ^+^ progenitor cells and CD4 ^+^ T cells express CXCR4, the receptor of this chemokine. This receptor plays a role in chemotaxis, invasion, angiogenesis, and proliferation. The role of the CXCR4/CXCL12 axis has been implicated in the pathogenesis of MF ^10^.

In CTCLs, malignant T cells display activation of the T cell receptor (TCR) pathway, which leads to TCR-dependent T helper 2 (Th2) cytokines such as IL-4 and IL-13 and resistance to natural mechanisms that prevent uncontrolled proliferation, such as Fas cell surface death receptor (FAS)-mediated apoptosis and transforming growth factor-beta-mediated growth suppression ^12^. Regulatory T cells with a CD4 ^+^ CD25 ^+^ phenotype comprise about 5% to 10% of peripheral T cells and play a role in tumor immunology. The role of these cells in CTCLs is controversial. Most studies have shown that, in CTCLs, a high count of FOXP3 ^+^ regulatory T cells is correlated with improved prognosis. This finding is completely opposite to that of studies on the role of these cells in solid tumors ^27^.

Studies have shown that CD26 is able to cleave and inactivate CXCL12; hence, its lack of expression in CTCLs results in enhanced CXCL12-dependent chemotaxis ^10^. On the other hand, CD164 is significantly overexpressed on CD4 ^+^ lymphocytes in SS. It seems that this factor can be a diagnostic parameter and a potential target for therapeutic approaches in SS ^39^. These are zinc-dependent enzymes implicated in gene regulation and in the modulation of numerous cellular pathways, including proliferation, differentiation, apoptosis, and migration. Aberration in the activity of these enzymes and their mutations has been reported in CTCLs ^1^.

In MF, the malignant cells migrate to the skin by using the ligand E-selectin on endothelial cells by the expression of a marker for skin homing, cutaneous lymphocyte-associated antigen (CLA). The ability of CLA to mediate leukocyte homing to the skin is dependent on specific chemokine receptor-ligand interactions. One of these interactions occurs through chemokine receptor CCR4; overexpression of this receptor has been reported in CTCL cases with peripheral blood involvement ^1^.

Granulysin is a cytotoxic, proinflammatory, and anti-microbial agent that is expressed along with granzymes and perforin in granules of cytotoxic T cells and natural killer cells. It plays a role in innate immunity, chemotaxis, and tumor immunology and has been shown to be involved in the progression of MF ^27^. Mucin 1 C-terminal subunit controls important pathways of oncogenesis by governing cell proliferation, self-renewal, tissue invasion, and apoptosis. This heterodimeric protein protects cells against reactive oxygen species-induced death. Overexpression of this protein has been shown in CTCL cell lines ^40^. It has been suggested that the maintenance of redox balance plays a critical factor in protecting malignant cells in the CTCLs from apoptosis ^40^.

Cancer stem cells have many similarities to normal stem cells, including infrequent division, high self-renewal capacity, resistance to apoptosis, and ability to maintain an undifferentiated state, overcome cellular senescence, and differentiate to all cell types. Because of their rare cell divisions, they are resistant to chemotherapeutic agents. Additionally, these cells are responsible for relapse and metastasis of tumors. The expression of embryonic stem cell genes such as Nanog homeobox ( *NANOG*), *SRY* (sex determining region Y)-box ( *SOX*)- *2*, and *OCT4* ( *POU* class 5 homeobox [ *POU5F*] *-1*) and their upstream and downstream signaling members was shown in CTCL lesions ^2^.

It was proposed that the formation of blood and lymphatic vasculature is involved in the progression of CTCL. Malignant T cells produce several angiogenic factors, such as podoplanin (PDPN), lymphatic vessel hyaluronan receptor-1 (LYVE-1), vascular endothelial growth factor-C (VEGF-C), VEGF-R3, and lymphotoxin alpha (LTα), which play roles in neoangiogenesis and neo-lymphoangiogenesis. The interaction of LTα, IL-6, and VEGF has been shown to induce angiogenesis by promoting endothelial cell sprouting and tube formation ^3^.

Some studies have reported an association between chronic cutaneous inflammation and subsequent development of CTCL ^9^. Chronic or professional exposure to topical chemical agents, long-lasting psoriasis, and urticaria have been proposed as risk factors ^21^. Chronically activated T lymphocytes may eventually result in the creation of an atypical T cell clone ^9^. For instance, in granulomatous MF, it appears the granulomatous inflammation may precede the lymphoma, resulting in lymphocyte proliferation through macrophage-produced IL-6 ^41^.

A relationship between microbial colonization/infection and MF has been suggested ^9, 42^. It has been shown that bacterial isolates containing staphylococcal enterotoxin-A (SEA) promote disease progression by inducing STAT3 activation and IL-17 expression in malignant T cells ^42^. On the other hand, during the evolutionary process of CTCLs, loss of the normal TCR repertoire results in immunosuppression and opportunistic infections leading to death ^12^.

The role of viral infection in the pathogenesis of the CTCLs remains controversial. Recently, the role of retroviruses such as human T cell leukemia virus type 1 (HTLV-1) and HTLV-2 and human immunodeficiency virus and herpesvirus family members like Epstein-Barr virus, human herpesvirus 8, and cytomegalovirus have been suggested in the pathogenesis of these disorders. Viral infection may promote tumoral infiltration by inducing the production of tumor necrosis factor-alpha (TNF-α), IL-6, and IL-1a in keratinocytes. Additionally, in the skin, these organisms play the role of a stable chronic antigen, which results in a clonal proliferation of T cells, leading to CTCLs ^4^.

## Diagnosis

The diagnosis of CTCLs is difficult at early stages because of the presence of multiple clinical presentations ^1, 43, 44^ and lack of definitive diagnostic criteria ^1, 45^. Hence, in most cases, it takes an average of 6 years from disease onset until confirmation of the diagnosis ^1, 44^.

Recently, there have been advances in the accurate diagnosis of CTCLs. To diagnose the CTCLs, guidelines prepared by the National Comprehensive Cancer Network recommend biopsy of suspicious skin sites and subsequent assessment in terms of dermatopathology, immunohistochemistry, and molecular analysis (TCR gene rearrangement) ^14^. Observation and palpation of the skin are mainstays in suspecting CTCLs. Palpation of lymph nodes remains the traditional approach for staging of these disorders ^14, 45, 46^. Frequently, many biopsies are required to make the definitive diagnosis, as morphologic and phenotypic manifestations of CTCLs are variable and information derived from a single biopsy can lead to misdiagnosis ^18, 19, 45, 46^. Identifying malignant cells in the peripheral blood of patients with CTCL is invaluable for detecting SS in early stages and determining prognosis ^47, 48^. However, blood analysis is of limited value because there is no precise marker in this analysis to detect the CTCLs in a sensitive way ^9, 14^. Lactate dehydrogenase (LDH) is a non-specific marker of tumor burden and is related to poor prognosis of CTCLs ^9, 14^. These studies provide a robust technique for assessing aberration of genes in the CTCLs ^3, 35^.

Detection of a malignant T cell clone is a critical marker for definite diagnosis of CTCLs. TCRγ polymerase chain reaction (PCR) analysis detects clones of T cells in only a subset of patients, whereas the sensitivity and specificity of high-throughput TCR sequencing to detect T cell clones are higher than in TCRγ PCR. Indeed, the technique of high-throughput TCR sequencing is useful for the accurate diagnosis of all stages of CTCLs, differentiation of these disorders from benign inflammatory disorders, and determination of origin and location of malignant CTCL cells ^14, 44^. Detection of malignant cells using flow cytometry in patients with SS is an important marker for diagnosing SS ^14, 39, 45^. In advanced cases, biopsies from bone marrow and lymph nodes are important factors in diagnosis ^14^.

The loss of cell surface markers such as CD26, CD27, and CD7 on malignant T cells is of value in diagnosing CTCLs. On the other hand, the overexpression of CD164 has been reported on CD4 ^+^ T cells of patients with SS. In flow cytometric studies, detection of more than 20% CD164 on CD4 ^+^ cells in the blood of erythrodermic cases is highly suspicious for SS. However, the sensitivity and specificity of these assays should be interpreted with caution ^48^. PCTCL-NOS is characterized by variable loss of almost all T cell antigens, CD56 positivity, and limited or absent CD30 expression ^8^. Ki67, CD34, and AgNORs are parameters of cell proliferation and angiogenesis that are well-known markers for progression of CTCLs. They are highly expressed in advanced stages of MF and are associated with shorter survival ^10^.

T-cell-specific soluble IL-2 receptor (sIL-2r) ^9^ is not specific for diagnosing CTCL but is a potential marker for activity, severity, and prognosis of this disorder. The association between increased sIL-2r and either adnexal disease or advanced-stage MF has been reported. This factor has better specificity as a prognostic factor than does LDH ^9^.

Recent studies have confirmed the role of the *TOX* gene as a disease marker of CTCLs. Moreover, this gene is a candidate for therapeutic targeting ^22^. Studies have suggested that *EPHA4* can be a diagnostic and prognostic marker for SS ^13^. miRNA profiling is a diagnostic marker for CTCLs. Studies have shown that minimal miRNA classifiers can determine malignant dermatoses ^25^.

HTLV serology should be considered in advanced cases ^14^. Magnetic resonance imaging (MRI) or computed tomography (CT) scan ^9^ is used to investigate nodal and systemic involvement ^9, 46^. Fluorine-18 fluorodeoxyglucose positron emission tomography-CT ( ^18^F-FDG PET-CT) ^46^ can determine cutaneous and extracutaneous lesions in CTCLs, response to therapy, and disease recurrence. In comparison with CT scan, this modality is more sensitive and specific in detecting both cutaneous and extracutaneous involvement, particularly in determining lymph node involvement ^46^.

## Management

There is no known cure for MF and SS ^11^; hence, therapeutic options are mostly palliative ^1, 49^, and the goals of treatment include relieving symptoms, inducing remission, and postponing progression while decreasing significant side effects caused by therapeutic modalities ^12^. Multi-drug therapeutic approaches are inappropriate for CTCLs because of the high risk of infection in patients with poor skin barrier ^50^.

Before choosing the best option for treating this malignant condition, accurate staging is essential ^1, 46, 51^. Generally, therapeutic options are classified into two groups. Skin-directed therapies ^1, 11, 45, 52, 53^ are the first choice for treating early stages of disease (IA to IIA) when involving less than 20% of the body surface ^1^. Systemic therapies ^1, 11, 45, 52, 53^ are used for refractory cases in the early stages and cases with advanced stages (at least ** IIB) ^1, 6^.

Corticosteroids in topical and systemic forms are effective in treating CTCLs ^9, 54, 55^. Topical corticosteroids can be employed for treating refractory cases of both early stage disease and more advanced cases. One of the problems with corticosteroid therapy is relapse of disease ^1^.

Retinoids are effective in treating the CTCLs through anti-proliferative and apoptosis-inducing effects ^56, 57^. Retinoic acid receptor β2 works as a tumor suppressor gene ^56^. Among topical retinoids, bexarotene, also known as Targretin, is approved by the US Food and Drug Administration (FDA) for treating stage I MF ^1, 58, 59^ and relapsed refractory CTCLs ^58^. Severe mixed hyperlipidemia with a significant decrease in high-density lipoprotein cholesterol level and central hypothyroidism are reversible, dose-dependent adverse effects of bexarotene ^60^. Tazarotene is another topical retinoid whose efficacy as a monotherapy has been shown in treating stages I to IIA CTCL ^61^. Systemic retinoids such as acitretin, isotretinoin, and bexarotene have been used successfully in the treatment of CTCLs ^9^.

HDACIs, classified as anti-neoplastic agents, are novel therapeutic options for treating CTCLs ^1, 9, 62^. Their mechanisms of action are via transcription-dependent and transcription-independent ways ^11^, including (a) promoting the expression of genes that regulate cell differentiation and apoptosis, (b) inducing changes to the structural integrity of chromatin ^1^, (c) regulating miR-22 expression ^25^, and (d) increasing the production of reactive oxygen species and decreasing mitochondrial membrane ^63^. These agents preferentially destroy transformed cells over normal cells ^11^.

In this therapeutic group, vorinostat ^1, 11, 50, 58^ and romidepsin ^1, 11, 50, 58, 63^ are FDA approved for treating progressive, persistent, or recurrent CTCLs. These agents, when given as single agents, can induce an overall response rate of 30% to 35%, but a complete response rate is seen in only 2% to 6% of cases ^11^. Entinostat, belinostat, panobinostat ^1^, AN-7 ^11^, and quisinostat ^64^ are other HDACIs currently under study. Generally, HDACIs are well tolerated. Fatigue, gastrointestinal discomfort, thrombocytopenia, neutropenia, anemia, and dehydration are insignificant side effects which have been reported with these agents ^1^.

Imiquimod is a Toll-like receptor 7 (TLR7) agonist that is effective in treating MF. It works through inducing the production of interferon-alpha (IFN-α), TNF-α, IL-1α, IL-6, and IL-8 from plasmacytoid dendritic cells, which are seen in inflamed and malignant skin lesions. The efficacy of topical resiquimod, an imidazoquinoline with TLR7- and TLR8-stimulating activity, was shown in treating early stage CTCLs. Its effectiveness in inducing regression of untreated lesions was reported, probably mediated by enhancing systemic anti-tumor immunity. Resiquimod appears to act by recruiting and expanding benign T cell clones, increasing skin T cell effector and natural killer cell functions ^54^.

Denileukin diftitox is a recombinant fusion protein ^9, 50^, composed of diphtheria toxin and IL-2, approved by the FDA for treating CTCLs ^51^.

The efficacy of zanolimumab and alemtuzumab in treating the CTCLs has been reported. Zanolimumab has a lower risk of infection than does alemtuzumab ^50^.

Cytokines such as IFN-α are effective in treating cases with MF and SS but can exacerbate PCTCL-NOS ^8^. IFN-α2b is still the best option as first-line systemic therapy for MF ^51^. Recombinant IL-12 is beneficial in treating CTCLs by inducing cellular immunity and cytotoxic T cell responses in the host ^54^.

Chemotherapeutic agents play a role in managing CTCLs, but severe adverse effects are reported ^11^. For the most part, topical chemotherapeutic agents such as mechlorethamine (nitrogen mustard) and carmustine are successful in managing early stage disorders, but their effectiveness in treating advanced cases is doubtful ^1, 11^. Mechlorethamine was approved by the FDA for the treatment of stage Ia and Ib MF. It is an alkylating agent, which acts by inhibiting proliferating cells and affecting keratinocyte-Langerhans cell–T cell interactions. Non-melanoma skin cancers have been seen in patients who have received this agent in combination with phototherapy, radiation, and immunosuppressive chemotherapy ^11^.

Other systemic chemotherapeutic agents that have been used to treat CTCLs include methotrexate, chlorambucil, gemcitabine, and pegylated doxorubicin ^9^. Pralatrexate is a methotrexate analog approved by the FDA for treating relapsed or refractory CTCLs ^50^. Studies have shown variable efficacy for cyclophosphamide, doxorubicin, vincristine, and prednisone (CHOP) in the treatment of advanced cases of CTCLs ^9, 50^.

Psoralen plus ultraviolet A (PUVA), ultraviolet B (UVB) ^9^, UVA1, and excimer laser ^65^ are among the most common treatments used for achieving remission or avoidance of progression in MF. In comparison with PUVA, UVB is less effective at treating infiltrated lesions; additionally, remission duration is shorter with UVB ^9^.

Radiotherapy is an effective skin-directed therapy for the treatment of CTCLs ^66, 67^. Lymphocytes are sensitive to radiation therapy. In more advanced cases, radiation therapy to local lesions or to the entire skin can control disease. For cases with a single lesion, this modality can be curative ^66^.

Electron beam radiation therapy is effective in treating CTCLs ^49, 50, 68, 69^ in stages I to III ^50^. Whole-body total skin electron beam is an appropriate modality for more advanced cases ^1, 50^. Complete response rate is lower in tumor-stage disease in comparison with plaque-stage cases (36% versus 98.3%) ^49^.

Conventional photodynamic therapy with aminolevulinic acid (ALA-PDT) is effective in a subset of CTCLs because it acts through apoptosis while the expression of death receptors like FAS in malignant T cells is low. The combination of methotrexate with ALA-PDT enhances the efficacy of photodynamic therapy through upregulating FAS by inhibiting the methylation of its promoter ^70^.

Extracorporeal photopheresis is an immunomodulating method resulting in the expansion of the peripheral blood dendritic cell population and enhancement of the TH1 immune response ^71^. It is an appropriate modality for treating refractory, early stage MF ^71^ and SS ^50, 71^. With this modality, a partial response rate of 30% to 80% and a complete remission rate of 14% to 25% are estimated ^50^.

Allogeneic hematopoietic stem cell transplantations are used for treating advanced stages of MF, SS ^7, 53, 54, 72, 73^, and PCTCL-NOS ^8^. Studies have shown that this therapeutic option is appropriate for young patients ^7, 50, 72^ with relapsing diseases which progress in spite of several lines of chemotherapy ^7^.

The efficacy of some agents, including tazarotene, lenalidomide, forodesine (BCX-1777), synthetic oligonucleotides, temozolomide, C-beta kinase inhibitor ^50^, mucin 1 C inhibitors ^40^, everolimus ^74^, PD1/PD-L1 inhibitors ^75^, brentuximab vedotin, and mogamulizumab, is under investigation ^62, 75^.

As mucin 1 is overexpressed in cells of CTCLs, therapies targeting mucin 1 are effective in treating CTCLs. Theoretically, mucin 1 C inhibitors (such as GO-203), which increase the level of reactive oxygen species and lead to oxidative-stress-induced late apoptosis/necrosis, can be an effective treatment ^40^. Everolimus, which targets the mammalian target of rapamycin (mTOR) pathway, appears to be effective in treating T cell lymphoma by inhibiting malignant T cell proliferation ^74^.

## Prognosis

CTCLs are lifelong disorders that recur after discontinuation of therapy, even in cases that do not progress ^54^. In spite of the introduction of several therapeutic options for CTCLs, as they progress and become refractory to treatment, the malignant cells have the propensity to infiltrate lymph nodes and peripheral blood vessels, resulting in debilitating states. Progression to tumor stage where the neoplastic cells spread to the lymph nodes and internal organs has been reported in less than 5% of cases with CTCL ^1^. In Table 3, prognostic factors for CTCLs are listed ^1, 5, 8, 9, 12, 14, 22, 27, 39, 40, 46, 51, 52, 76, 77^.

**Table 3.** Prognostic factors in cutaneous T cell lymphoma cases.

**Clinical factors** Age ^5, 8, 52, 77^ Sex ^52^ Staging ^47, 52, 53^ Extent and type of cutaneous involvement ^1, 9, 47, 53^ Presence of extracutaneous involvement ^1, 5, 46, 52^ such as blood ^1, 5, 52, 53^, bone marrow ^8^, and adnexal ^9^ involvement Disease progression ^1^ Old refractoriness to successive treatment protocols ^5^
**Laboratory factors** Proliferation index ^52^ Folliculotropism ^52^ Presence of Sézary cells ^5, 40^ Large cell transformation in histology ^14, 41, 52^ White blood cell/lymphocyte count ^52^ Loss of T cell receptor repertoire ^39^ Increased level of soluble interleukin-2 receptor at initial diagnosis ^9^ Normal or high levels of lactate dehydrogenase ^8, 52, 77^ CD30 expression of less than 10% ^52, 77^ Overexpression of TOX ^22^ Expression of proliferation markers Ki-67, MCM-3, and MCM-7 ^78^ MicroRNA profiling ^25^ Granulysin expression ^27^ Presence of FOXP3 ^+^ regulatory T cells ^27^ EPHA4 expression ^13^

In CTCL cases with visceral involvement, skin lesions are severe and the risk of skin infection is high. On the other hand, autopsy studies have shown that between 70% and 90% of patients with MF die with visceral involvement ^5^. Moreover, primary CTCLs have distinctive clinical behavior in comparison with systemic lymphomas with skin involvement ^78^.

At early stages, there is no significant difference between the life expectancy of patients with CTCL and that of healthy people ^1, 5^, whereas in more progressed cases, life expectancy decreases to 3.2 to 9.9 years ^1^.

Patients with MF have a chronic course lasting from years to decades; many of them die from unrelated disorders, whereas about 25% of them die of lymphoma ^9^. Immunosuppression and opportunistic infections are the most common causes of disease-related death ^11^. The prognosis of SS is poor. Its median survival rate is from 2 to 4 years ^1^, and its 5-year survival rate is approximately 18% to 20% ^7^. In PCTCL-NOSs, the 5-year survival rate is less than 20% ^8^.

## Conclusion

Although there are many studies regarding the pathogenesis and management of the CTCLs, many questions about this complicated group of diseases remain unanswered.
